# Effects of a blocked versus an alternated sequence of balance and plyometric training on physical performance in youth soccer players

**DOI:** 10.1186/s13102-019-0131-y

**Published:** 2019-09-02

**Authors:** Thomas Muehlbauer, Vincent Wagner, Dennis Brueckner, Simon Schedler, Gerrit Schwiertz, Rainer Kiss, Marco Hagen

**Affiliations:** 1 Division of Movement and Training Sciences/Biomechanics of Sport, University of Duisburg-Essen, Gladbecker Str. 182, 45141 Essen, Germany; 2 Department of Health and Social Affairs, FHM Bielefeld - University of Applied Sciences, Bielefeld, Germany

**Keywords:** Postural control, Muscle power, Speed, Agility, Adolescence

## Abstract

**Background:**

The sequence of blocked balance training (BT) followed by blocked plyometric training (PT) showed greater improvements in physical performance than vice versa and is explained by a preconditioning effect of BT-related adaptations on subsequent adaptations induced by PT. However, it remains unclear whether beneficial effects can also be induced using alternating instead of blocked BT and PT exercise sequences. Thus, we examined the effects of a blocked versus an alternated sequence of BT and PT on physical performance in trained individuals.

**Methods:**

Twenty young soccer players (13 years) were randomly assigned to a blocked (*n* = 10) or an alternated (*n* = 10) intervention group. Both groups trained balance and plyometric exercises for six weeks (two sessions/week). The exercises were conducted in a blocked (three weeks of BT followed by three weeks of PT) or an alternated sequence (weekly change of BT and PT). Assessment of pre- and post-training performance included measures of balance, muscle power, speed, and agility.

**Results:**

Mainly significant main effects of Test (i.e., pre- to post-test improvements) were observed for the Y-balance test (*p* ≤ 0.014, 1.3 ≤ Cohen’s *d* ≤ 1.81), the squat jump (*p* = 0.029, *d* = 1.36), the countermovement jump (*p* = 0.002, *d* = 2.21), the drop jump (*p* = 0.004, *d* = 1.96), the split times/total time over 15-m sprinting (*p* ≤ 0.001, 2.02 ≤ *d* ≤ 3.08), and the figure-T agility run (*p* < 0.001, *d* = 3.80). Further, tendencies toward significant Test x Group interactions were found for several items of the Y-balance test and for SJ height in favor of the blocked BTPT group.

**Conclusions:**

Our results indicate that the combined training of balance and plyometric exercises is effective to improve proxies of physical performance in youth soccer players. In addition, there is a limited advantage in some parameters of balance and muscle power for the blocked as compared to the alternated sequence of BT and PT.

## Background

Recent long-term athletic development models showed that, in youth athletes, balance and muscle strength/power exercises should be integrated into the training regimes over long periods of time and starting from the early beginning of an athletes career (i.e., during childhood/adolescence) [1, 2]. Previous studies [3, 4] on the effects of single-mode balance training (BT) or plyometric training (PT) on physical performance in trained youth showed significant improvements in most of the investigated variables. For example, Heleno et al. [4] conducted a five-week BT and soccer training in young male players (age range: 14–16 years). Compared to an active control group (i.e., soccer training only), the intervention group significantly improved measures of balance (e.g., Star excursion balance test) and muscle power (e.g., side hop test). In terms of PT, Ramirez-Campillo et al. [3] investigated the effects of different jump exercise programs (i.e., vertical jumps only, horizontal jumps only, combination of vertical and horizontal jumps) in young soccer players (age range: 10–14 years). In comparison to the active control group (i.e., soccer training only), all PT programs resulted in significant improvements in muscle power (e.g., countermovement jump [CMJ]) and balance (e.g., two-legged stance).

The manifold physical demands during soccer (i.e., strength as well as endurance-related aspects of physical performance) require a multimodal training approach [5]. However, time in training for youth athletes is limited and has to be shared with other everyday activities (e.g., school, leisure-time activities) and coaches are advised to effectively combine different training modalities. In this regard, the combination of balance and muscle strength/power exercises seems to be an appropriate possibility, since both modalities induced adaptation on the neuromuscular level [6, 7]. For example, Bruhn et al. [8] were able to show that (i) a blocked combination of balance and muscle strength exercises effectively improved physical performance, and (ii) improvements in performance were more pronounced if balance exercises were followed by strength exercises than vice versa. In another study, Hammami and colleagues [9] replicated these findings using a sequenced combination of balance and plyometric instead of balance and strength exercises. Both studies showed that a blocked sequence of BT followed by a blocked sequence of muscle strength/power training was more effective compared to the vice versa sequence of training. Hammami et al. [9] explained their findings by preconditioning effects of BT-related adaptations on subsequent adaptations induced by PT. However, it remains unclear and there is still a void in the literature on whether the positive effects of BT/PT training can be initiated early on using an alternating training sequence (i.e., weekly change of BT/PT) instead of a blocked one (i.e., several weeks of BT followed by several weeks of PT). In fact, there is evidence that preconditioning effects of BT-related adaptations on subsequent adaptations induced by PT are initiated early on and consequently, an alternating order might be more efficient compared to blocked schedule. On the one hand, Chaouachi et al. [10] showed significant improvements in physical performances of youth soccer players (age range: 13–14 years) when balance and plyometric exercises were combined within each training session. On the other hand, underlying mechanisms indicate that BT leads to (early) neuromuscular adaptations (e.g., improved activation of muscles encompassing the ankle joint) [11], representing a muscle group that is predominantly involved in proper jumping performance.

Thus, the aim of the present study was to investigate the effects of an alternated versus a blocked sequence of BT and PT on physical performance in young soccer players. With reference to the relevant literature [8, 9], we expected that both exercise conditions will result in enhanced performance but the improvement will be larger in the alternated balance and plyometric exercise (BTPT) group as in the blocked BTPT group.

## Methods

### Participants

Twenty young, sub-elite, male soccer players (13 years) volunteered to participate in this study. The players were member of a second division German soccer club. After pre-testing, the participants were randomly assigned to a blocked (*n* = 10) or an alternated (*n* = 10) BTPT group. All but one player of the blocked BTPT group (due to common cold) received treatment conditions as initially allocated. No test-related injury was detected but two players (i.e., one of each group) reported a competition-related injury. Thus, performance data of 17 players (blocked BTPT: *n* = 8, alternated BTPT: *n =* 9) were used for the analyses. The characteristics of the study participants are shown in Table 1 with no significant differences between the two intervention groups. All players trained four times per week (385 min/week) and played one match during the weekend. Participants’ assent and parents’ written informed consent were obtained prior to the start of the study. The Human Ethics Committee at the University of Duisburg-Essen, Faculty of Educational Sciences approved the study protocol.

**Table 1:** Characteristics of the study participants by intervention group

Characteristic	Blocked BTPT (*n* = 8)	Alternated BTPT (*n* = 9)	*p*-value
Age [years]	13.0 ± 0.9	13.1 ± 1.0	.990
Body mass [kg]	53.2 ± 8.7	52.4 ± 10.2	.860
Body height [cm]	166.3 ± 5.9	164.2 ± 9.5	.555
BMI [kg/m^2^]	19.1 ± 2.3	19.3 ± 1.9	.910
Maturity offset^a^ [years from PHV]	−0.2 ± 0.3	−0.3 ± 0.4	.555
Dominant leg length^b^ [cm]	102.5 ± 4.1	99.6 ± 9.2	.374
Non-dominant leg length [cm]	102.1 ± 4.1	99.5 ± 8.8	.398
Training volume [min/week]	385	385	

Values are mean values ± standard deviations. ^a^Maturity offset was calculated by using the formula provided by Moore et al. [12]. ^b^Leg dominance was determined according to the lateral preference inventory provided by Coren [13]. *BMI* body mass index, *BTPT* balance and plyometric training, *PHV* peak height velocity

### Soccer, balance and plyometric training program

The study was conducted between the first and second half of the soccer season. Both exercise groups conducted six weeks of soccer-specific training (four times a week) that included sprint, agility, and strength exercises as well as technical and tactical drills. The sessions were scheduled on Monday (115 min), Tuesday (100 min), Thursday (90 min), and Friday (80 min). In two of those sessions, the players either performed a blocked (Table 2) or an alternated (Table 3) BTPT program. The blocked BTPT group performed three weeks of balance followed by three weeks of plyometric exercises. The alternated BTPT group conducted the same exercises but changed the BT and PT program on a weekly basis. Both programs lasted between 15 to 26 min and were performed after a 15-min warm-up program including submaximal running (e.g., skipping, hip in/out), balance (e.g., forward/backward beam walking, single leg stance on unstable devices), and plyometric (e.g., submaximal multidirectional jumps) exercises. Progression during training was achieved by means of increasing the number of repetitions and exercise duration and difficulty of the balance/jump tasks. All sessions ended with a 15-min cool-down that included flexibility exercises and jogging at light intensity.

**Table 2:** Description of the blocked balance and plyometric training program

Exercise	Week 1	Week 2	Week 3	Week 4	Week 5	Week 6
*Balance*						
One-legged stance on an Airex® balance pad	3 × 30 s	3 × 40 s	3 × 40 s	–	–	–
One-legged stance on a Vitality® stability trainer	3 × 30 s Level 3	3 × 40 s Level 5	3 × 40 s Level 6	–	–	–
One-legged stance on an ankle disk with eyes closed	3 × 30 s	3 × 40 s	3 × 40 s	–	–	–
3-m backward beam walk	1 × 4 4.5 cm beam width	1 × 4 3.0 cm beam width	1 × 6 3.0 cm beam width	–	–	–
*Plyometric*						
Ankle jumps	–	–	–	2 × 8	2 × 10	2 × 12
Squat jumps	–	–	–	1 × 8	1 × 10	1 × 12
Skater jumps				2 × 6	2 × 8	2 × 10
One-legged rebounding jumps	–	–	–	2 × 4/leg	2 × 5/leg	2 × 6/leg
Split squats	–	–	–	2 × 4/leg	2 × 6/leg	2 × 8/leg
Total practice time	22 min.	24 min.	26 min.	15 min.	20 min.	25 min.
Total ground contacts	–	–	–	68	90	112

Values are sets by repetitions or exercise duration

**Table 3:** Description of the alternated balance and plyometric training program

Exercise	Week 1	Week 2	Week 3	Week 4	Week 5	Week 6
*Balance*						
One-legged stance on an Airex® balance pad	3 × 30 s	–	3 × 40 s	–	3 × 40 s	–
One-legged stance on a Vitality® stability trainer	3 × 30 s Level 3	–	3 × 40 s Level 5	–	3 × 40 s Level 6	–
One-legged stance on an ankle disk with eyes closed	3 × 30 s	–	3 × 40 s	–	3 × 40 s	–
3-m backward beam walk	1 × 4 4.5 cm beam width		1 × 4 3.0 cm beam width		1 × 6 3.0 cm beam width	
*Plyometric*						
Ankle jumps	–	2 × 8	–	2 × 10	–	2 × 12
Squat jumps	–	1 × 8	–	1 × 10	–	1 × 12
Skater jumps		2 × 6		2 × 8		2 × 10
One-legged rebounding jumps	–	2 × 4/leg	–	2 × 5/leg	–	2 × 6/leg
Split squats	–	2 × 4/leg	–	2 × 6/leg	–	2 × 8/leg
Total practice time	22 min.	15 min.	24 min.	20 min.	26 min.	25 min.
Total ground contacts	–	68	–	90	–	112

Values are sets by repetitions or exercise duration

### Testing procedures

Both, the pre- and post-testing was conducted in the afternoon (i.e., from 4 to 6 pm) using the same gym and by the same skilled assessors (graduated sport scientists). All players received standardized verbal instructions and a visual demonstration regarding the testing procedure that included assessment of anthropometric variables, dynamic balance (Y-Balance test), lower-extremity muscle power (squat [SJ], countermovement, and drop jump [DJ]), maximal speed (15-m sprint), and agility (figure-T run). This sequence of measurements was the same during the pre- and post-testings. Prior to each testing, all players conducted a standardized warm-up, which consisted of balance exercises, submaximal plyometric exercises, short linear sprints, and change-of-direction sprints.

#### Assessment of balance

The Y-Balance test was performed using the Test Kit (Functional Movement Systems®, Chatham, USA) that consists of a centralized stance platform to which three pipes are attached that represent the anterior (AT), posteromedial (PM), and posterolateral (PL) reach directions. Each pipe is marked in 0.5-cm increments for measurement purposes and equipped with a moveable reach indicator. Before the test started, the respective lengths of the participants’ right and left legs were determined in supine position by measuring the distance from the anterior superior iliac spine to the most distal aspect of the medial malleolus [14]. Afterwards, each participant was instructed to reach with one leg as far as possible while maintaining his balance in the AT, PM, and PL directions while standing without shoes on the centralized stance platform. Each player performed three practice trials to become familiar with the task, followed by three data-collection trials. As recommended by Plisky et al. [14], participants started with the right leg placed behind the red starting line of the stance platform and the left leg touching and moving the reach indicator with the most distal part of the foot in the AT direction. Afterwards, the participants returned to a bipedal stance position. The same procedure was conducted while standing on the left leg and reaching with the right leg. This sequence was repeated for the PM and PL reaches. An experienced examiner documented the distance from the center of the stance platform to the maximal reach indicator position after each reach to the nearest 0.5 cm. Trials were discarded and repeated until a total of three valid trials were achieved if the participant: a) had lost its balance at any point during the trial, b) had lifted the stance leg from the stance platform, c) had stepped on top of the reach indicator for support, or d) kicked the reach indicator. For further analyses, the normalized maximal reach distance per reach direction and leg was calculated as follows: normalized maximal reach distance (% leg length [LL]) = (absolute maximal reach distance [cm]) / LL [cm]) × 100. Additionally, the normalized composite score (CS) per leg was calculated by using the following formula: CS (% LL) = ((AT + PM + PL) / (LL × 3)) × 100.

#### Assessment of muscle power

Performance in three vertical jump tests (SJ, CMJ, DJ) were assessed using the OptoJump® system (Microgate Srl, Bolzano, Italy). For each jump test, participants were instructed to stand with feet shoulder width apart and to place their hands on hips. During the SJ, players were asked to perform a fast upward movement from a squatted position and to jump as high as possible. For the CMJ, participants were instructed to perform a downward movement as quickly as possible from an upright standing position to a self-selected depth, which was followed immediately by a maximal vertical jump. During the DJ, the participants stood on a box (drop height: 40 cm) and were asked to step off the box, drop down, land with both feet and jump off the ground as fast and as high as possible. The quality of the respective jump technique was controlled through visual on-site inspection of a skilled experimenter (graduated sport scientist). Each player performed three practice trials to become familiar with the task, followed by three data-collection trials. The resting period between trials amounted to approximately two minutes. The best SJ, CMJ, and DJ trial in terms of maximal jump height was taken for further data analysis.

#### Assessment of speed

Speed performance was assessed by means of the 15-m linear sprint test. Four double-light barriers (Witty®, Microgate Srl, Bolzano, Italy) were used for the detection of total time (i.e., 0–15 m) and split times (i.e., 0–5 m, 0–10 m). Participants were asked to begin the test with one foot positioned at the starting line in frontal erect position. The measurement started when the player passed the first double-light barrier. No starting signal was provided so that the subjects were able to individually start the sprint test. Participants were instructed to run as fast as possible. Each participant performed two trials with a 2-min rest between trials. The best trial (i.e., lowest sprinting time) was used for further analysis.

#### Assessment of agility

Agility performance was determined using the figure-T run test. Using one double-light barrier (Witty®, Microgate Srl, Bolzano, Italy), the participants were instructed to run in different techniques (i.e., forward, shuffling, backward) a figure-T course as fast as possible. Similar to the 15-m linear sprint test, no starting signal was provided so that the athletes were able to individually start the run. Two trials were performed by each player, and the trial with the shortest time was used for further analysis. The rest between trials was 5 min.

### Statistical analyses

Descriptive data are reported as group mean values and standard deviations after normal distribution was examined by the Shapiro-Wilk test (*p* > 0.05). An univariate analysis of variance (ANOVA) was used to test for significant differences in pre-testing values between the two groups. Afterwards, a 2 (Test: pre, post) × 2 (Group: blocked BTPT, alternated BTPT) ANOVA with repeated measures on Test was used. Furthermore, differences between pre- and post-test values were analysed for each group separately using paired *t*-tests. Further, effect sizes were calculated by converting partial eta-squared to Cohen’s *d*. According to Cohen [15], *d* = 0.2 represent small effects, *d* = 0.5 represent moderate effects, and *d* = 0.8 represent large effects. All statistical analyses were performed using Statistical Package for Social Sciences version 24.0. The significance level was set at *p* < 0.05. Tendencies toward significance were denoted as 0.05 ≤ *p* < 0.10.

## Results

Table 4 displays statistics for all analyzed variables. Generally, there were no statistically significant differences in pre-test values between the two intervention groups. Further, the attendance rates during training sessions amounted to 92 and 87% in the blocked and alternated BTPT group, respectively.

**Table 4:** Effects of balance and plyometric training (BTPT) on measures of physical performance in youth soccer players by intervention group

Variables	Blocked BTPT (*n* = 8)			Alternated BTPT (*n* = 9)			*p*-value (Cohen’s *d*)		
	Pre	Post	∆%^a^	Pre	Post	∆%^a^	Test	Test x Group	Group
*Balance*									
AT-ND [% LL]	65.9 ± 8.2	67.4 ± 4.4	2.3	72.7 ± 11.1	73.0 ± 10.7	0.4	.418 (.39)	.563 (.29)	.142 (.75)
PM-ND [% LL]	99.8 ± 3.4	105.6 ± 6.8	5.8	104.3 ± 7.5	106.0 ± 8.2	1.6	.006 (1.54)	.101 (.84)	.400 (.42)
PL-ND [% LL]	97.1 ± 5.3	104.3 ± 5.4	7.4	103.1 ± 9.3	105.4 ± 11.1	2.2	.002 (1.78)	.076 (.92)	.339 (.48)
CS-ND [% LL]	86.5 ± 7.5	91.2 ± 7.9	5.4	95.1 ± 16.3	96.6 ± 17.0	1.6	.002 (1.81)	.067 (.95)	.257 (.57)
AT-D [% LL]	66.0 ± 5.9	66.0 ± 5.7	0	70.3 ± 9.8	73.0 ± 11.1	3.8	.344 (.49)	.343 (.49)	.172 (.71)
PM-D [% LL]	99.9 ± 4.4	106.4 ± 9.3	6.5	103.4 ± 7.7	106.8 ± 11.2	3.3	.014 (1.38)	.425 (.41)	.604 (.26)
PL-D [% LL]	97.9 ± 7.1	104.6 ± 10.8	6.8	100.7 ± 8.1	107.3 ± 14.6	6.6	.009 (1.48)	.984 (.01)	.557 (.30)
CS-D [% LL]	86.5 ± 7.4	90.8 ± 9.7	5.0	93.1 ± 15.4	97.7 ± 20.3	4.9	.014 (1.38)	.929 (.06)	.334 (.50)
*Muscle power*									
SJ height [cm]	24.5 ± 3.3	26.7 ± 3.6	9.0	25.0 ± 3.9	25.3 ± 3.6	1.2	.029 (1.36)	.098 (.99)	.803 (.14)
CMJ height [cm]	25.5 ± 3.6	28.1 ± 3.4	10.2	26.2 ± 4.3	28.1 ± 3.3	7.3	.002 (2.21)	.573 (.32)	.857 (.11)
DJ height [cm]	22.0 ± 3.9	27.0 ± 3.6	22.7	23.7 ± 1.5	25.9 ± 3.2	9.3	.004 (1.96)	.209 (.74)	.807 (.14)
*Speed*									
0–5 m time [s]	1.21 ± 0.05	1.14 ± 0.07	5.8	1.21 ± 0.10	1.12 ± 0.03	7.4	.001 (2.02)	.538 (.33)	680 (.22)
0–10 m time [s]	2.04 ± 0.06	1.94 ± 0.09	4.9	2.02 ± 0.12	1.88 ± 0.06	6.9	<.001 (3.08)	.331 (.52)	.309 (.54)
0–15 m time [s]	2.77 ± 0.08	2.66 ± 0.13	4.0	2.75 ± 0.16	2.60 ± 0.09	5.5	<.001 (2.66)	.431 (.42)	.441 (.41)
*Agility*									
T test time [s]	10.67 ± 0.53	10.07 ± 0.39	5.6	10.46 ± 0.46	9.99 ± 0.32	4.5	<.001 (3.80)	.396 (.45)	.462 (.39)

Values are mean values ± standard deviations. ^a^Positive values indicate performance improvements. Figures in brackets are effect sizes (Cohen’s *d*) with 0 ≤ *d* ≤ 0.49 indicating small, 0.50 ≤ *d* ≤ 0.79 medium, and *d* ≥ 0.80 large effects. *AT* anterior, *CS* composite score, *CMJ* countermovement jump, *D* dominant leg (i.e., kicking leg), *DJ* drop jump, *LL* leg length, *ND* non-dominant leg (i.e., stance leg), *PL* posterolateral, *PM* posteromedial, *SJ* squat jump

### Balance

Irrespective of leg (i.e., non-dominant or dominant), the analyses revealed statistically significant main effects of Test for all but one (i.e., AT reach direction) reach directions (7.573 ≤ *F*_1, 15_ ≤ 13.833, *p* ≤ 0.014, 1.3 ≤ Cohen’s *d* ≤ 1.81). Further, trends toward significant Test × Group interactions were found for the PL reach direction (*F*_1, 32_ = 3.570, *p* = 0.076, Cohen’s *d* = 0.92) and the CS (*F*_1, 32_ = 3.822, *p* = 0.067, Cohen’s *d* = 0.95) of the non-dominant leg. Post-hoc analyses revealed significant medium- to large-sized improvements from pre- to post-test in the blocked (PL-ND: Δ7.4%, *p* = 0.002, Cohen’s *d* = 1.34; CS-ND: Δ5.4%, *p* = 0.004, Cohen’s *d* = 0.62) but not in the alternated (PL-ND: Δ2.2%, *p* = 0.272, Cohen’s *d* = 0.22; CS-ND: Δ1.6%, *p* = 0.244, Cohen’s *d* = 0.09) BTPT group. In addition, no significant main effects of Group were observed for all measures of balance performance (Table 4).

### Muscle power

The statistical analysis showed significant main effects of Test for SJ height (*F*_1, 15_ = 6.000, *p* = 0.029, Cohen’s *d* = 1.36), CMJ height (*F*_1, 15_ = 15.802, *p* = 0.002, Cohen’s *d* = 2.21), and DJ height (*F*_1, 15_ = 12.485, *p* = 0.004, Cohen’s *d* = 1.96). In addition, a tendency toward a significant interaction effect of Test × Group was found for SJ height (*F*_1, 32_ = 3.178, *p* = 0.098, Cohen’s *d* = 0.99). Post-hoc analyses yielded significant medium-sized improvements from pre- to post-test in the blocked (Δ9.0%, *p* = 0.010, Cohen’s *d* = 0.63) but not in the alternated (Δ1.2%, *p* = 0.686, Cohen’s *d* = 0.09) BTPT group. No significant main effects of Group were detected for any of the investigated jump variables (Table 4).

### Speed

Statistically significant main effects of Test were detected for all measures of speed performance (15.232 ≤ *F*_1, 15_ ≤ 35.715, *p* ≤ 0.001, 2.02 ≤ Cohen’s *d* ≤ 3.08). No significant main effects of Group nor Group × Test interactions were found (Table 4).

### Agility

A statistically significant main effect of Test was shown for the figure-T run (*F*_1, 15_ = 53.969, *p* < 0.001, Cohen’s *d* = 3.80). No significant main effect of Group nor a Group × Test interaction was observed (Table 4).

## Discussion

We investigated the effects of a blocked versus an alternated sequence of balance and plyometric exercises on physical performance in trained individuals. In this regard, we assessed proxies of balance, muscle power, speed, and agility performance in young, sub-elite, male soccer players who either conducted six weeks of BTPT in a blocked (i.e., 3 weeks of BT + 3 weeks of PT) or an alternated (i.e., weekly change of BT and PT) order. The main findings of this study can be summarized as follows: (1) in both groups, the majority of physical performance measures (i.e., Y-balance test reach distances, SJ, CMJ, DJ height, split times/total time over 15-m sprinting, figure-T run time) were enhanced after the training period; (2) the performance improvements in some parameters of balance (i.e., PL reach distance and CS of the non-dominant leg) and leg muscle power (i.e., SJ height) were medium to large for the blocked and small for the alternated BTPT group.

### Combinatory effects of balance and plyometric training

In accordance with our hypothesis, we found that both exercise conditions resulted in enhanced physical performance. This finding fits well with the results from earlier studies combining balance and plyometric exercises. For example, Chaouachi et al. [16] assigned adolescent boys (age range: 12–15 years) to a combined BT and PT group, a single-mode PT group or a passive control group. After eight weeks of training (3 sessions per week), both training groups improved their balance, muscle power/strength, speed, and agility performances as compared to the control group. In addition, the combined group showed larger improvements than the single-mode group in measures of muscle power, speed, and agility. Further, Bouteraa et al. [17] investigated the effect of eight weeks (2 times per week) of combined BT and PT on proxies of physical fitness in female adolescent basketball players (mean age: ~ 16 years). In comparison to the active control group (i.e., basketball training only), the combined group enhanced their balance, muscle power, and agility performance. The findings of the present study and those of the current literature [16, 17] suggest that the combination of balance and plyometric training is suitable to improve physical performance in youth athletes. Further and based on the calculated maturity offset (Table 1), we investigated a population of trained individuals in the middle of their growth spurt. This is a time when youth might experience adolescent awkwardness [18] and when some injuries (e.g., soft tissue) in soccer increase [19]. Showing that players in their growth spurt can improve their physical performances in response to the applied training regimens is a noteworthy finding for coaches that are involved in training young athletes.

### Sequencing effects of balance and plyometric training

There is empirical evidence [8, 9] that the order of balance and strength/plyometric exercises differentially affects improvements in physical fitness. More precisely, a sequence of blocked BT followed by blocked PT resulted in greater enhancements than the opposite order. In this regard, Hammami et al. [9] trained 12- to 13-year-old soccer players for eight weeks (2 sessions per week) with either an initial four weeks of BT followed by another four weeks of PT or vice versa. They found that BT at first and PT afterwards resulted in either similar or superior (in 5 out of 13 outcomes) physical performance improvements compared with the other way around (i.e., PT first, BT afterwards). Further, Chaouachi et al. [10] investigated young male soccer players (age range: 13–14 years) that performed balance and plyometric exercises within each training session as alternating pairs or in a blocked fashion (balance before plyometrics). Following eight weeks of training (2 sessions per week), they observed that both groups significantly improved their balance, muscle strength/power, speed, and agility performances. Hammami et al. [9] explained the BT-related adaptations on subsequent adaptations induced by plyometric exercises by a so-called “preconditioning effect”. On a behavioral level, BT leads to decreased body sway and increased postural stability while standing [20], representing a prerequisite for well-developed and trained jumping as well as landing performance. Further, BT has been shown to significantly improve postural control in various cohorts [21]. By this means, variations in the axial direction of ground reaction forces that may hamper jumping performance (i.e., potential misalignments of force vectors during jumping) can be reduced and the generation of force can be improved. On a neuromuscular level, BT results in an improved activation of muscles that encompass the ankle joint [11], representing muscle groups predominantly involved in proper and safe jumping performance.

With the goal to enlarge this beneficial effect, we alternated balance with plyometric exercises on a weekly basis and compared the effects with those generated by a blocked sequence of three weeks of BT followed by another three weeks of PT. Contrary to our assumption, greater performance improvements were detected for the blocked (i.e., medium- to large-sized effects) compared to the alternated (small-sized effects) BTPT group in some measures of balance and muscle power. Interference effects might explain our finding of greater improvements from pre- to post-testing in the blocked BTPT group. More specifically, a weekly change of BT with PT sessions seems to interrupt the specific BT- versus PT-related adaptations. Although both training regimens (BT and PT) induce changes on the neuromuscular level [6–8], adaptations seem to be training- and/or task-specific. That is, four weeks of BT resulted in reduced cortico-spinal excitability while four weeks of ballistic strength training resulted in enhanced cortico-spinal activation [22]. Three weeks of blocked BT followed by three weeks of blocked PT training seem to be suitable to reduce interfering effects of neural adaptation patterns leading to facilitating the trainings effects instead. In fact, Gruber et al. [23] were able to show that four weeks of BT significantly improved postural control (i.e., less sway) and muscle activation (i.e., reduced EMG amplitude) in a training compared to a control group. Taken together, the results of the present study and those of previous work [9] imply that the sequence of BT and PT differentially affects physical fitness improvements in youth athletes. More precisely, if the goal is to enhance measures of physical performance in young athletes, coaches will be advised to use a blocked sequence of BT followed by PT instead of the opposite or a weekly alternated order.

Our study includes four limitations that need to be addressed. First, our sample size is relatively small, which will have influenced the statistical power and ability to detect significant effects. Second, our findings are limited to the examined age group (i.e., 13-year-olds). Therefore, we cannot comment on training-related adaptations to other groups of younger or older soccer players. Third, we did not include a control group. However, the inclusion of a passive control group (i.e., no training) would be difficult in an athletic setting, as we cannot expect young, sub-elite soccer players to stop their training for six weeks. Also, the enclosure of an active control group (i.e., soccer training only) is hardly practicable, because balance and plyometric exercises are important components of the regular soccer training in youth players [24]. Thus, the observed improvements in physical performance are most likely the result of soccer-specific adaptations (including sprint, agility, and strength exercises) and alterations due to the applied balance and plyometric exercises. Fourth, the underlying mechanisms of the training-induced improvements in physical performance remain unclear, as our methodological approach was limited to behavioral outcome measures.

## Conclusions

The present study investigated the effects of a blocked versus an alternated sequence of balance and plyometric exercises on physical performance in young, sub-elite, male soccer players. We found that both sequences of BTPT yielded improvements in measures of balance, muscle power, speed, and agility performance. Additionally, the blocked BTPT group showed medium- to large-sized and the alternated BTPT group displayed small-sized improvements from pre- to post-testing in some parameters of balance and muscle power. Thus, it is recommended to use a blocked rather than an alternated sequence of balance and plyometric exercises if the goal is to improve proxies of physical performance in a superior extent.

## Data Availability

The data generated and analysed during the present study are not publicly available due to ethical restrictions but are available from the corresponding author upon reasonable request.

## Abbreviations

ANOVA: Analysis of variance
AT: Anterior
BMI: Body mass index
BT: Balance training
BTPT: Balance and plyometric exercise
CMJ: Countermovement jump
CS: Composite score
D: Dominant
DJ: Drop jump
EMG: Electromyography
LL: Leg length
ND: Non-dominant
PHV: Peak height velocity
PL: Posterolateral
PM: Posteromedial
PT: Plyometric training
SJ: Squat jump
